# IL12p40 Regulates Functional Development of Human CD4^+^ T Cells: Enlightenment by the Elevated Expressions of IL12p40 in Patients With Inflammatory Bowel Diseases

**DOI:** 10.1097/MD.0000000000000613

**Published:** 2015-03-13

**Authors:** Xiaobing Wang, Ting Wu, Feng Zhou, Shi Liu, Rui Zhou, Siying Zhu, Lu Song, Feng Zhu, Ge Wang, Bing Xia

**Affiliations:** From the Department of Gastroenterology/Hepatology (XW, TW, F Zhou, SL, RZ, SZ, LS, F Zhu, GW, BX), Zhongnan Hospital of Wuhan University; and The Hubei Clinical Center & Key Laboratory of Intestinal & Colorectal Diseases (XW, TW, SL, RZ, SZ, LS, F Zhu, GW, BX), Wuhan, Hubei, PR China.

## Abstract

The proinflammatory effects of IL12p40 had been documented in the literature, and anti-IL12p40 treatment had been proved to be effective in therapy of Crohn disease (CD) in a phase 2b clinical trial. However, the precise role of IL12p40 in the pathogenesis of inflammatory bowel disease (IBD) was still poorly understood. In this study, we investigated the expressions of IL12p40 and its receptor interleukin-12 receptor β 1 both locally and systemically in IBD cases and healthy controls, and the contribution of IL12p40 in IBD pathogenesis. We found that the expression of IL12p40 was elevated both at messenger RNA and protein levels systematically and locally in IBD patients but more significantly in CD patients. Our genetic association study revealed that the polymorphisms of IL12B rs6887695 were associated with both CD and ulcerative colitis (UC) susceptibility in Chinese population, but did not affect the serum IL12p40 level in either CD patients or UC patients. In addition, CD4^+^ T cells isolated from peripheral blood of CD patients secreted the most abundant IL12p40 production, compared with the UC patients and healthy controls. We also found for the first time that neutralizing IL12p40 secretion could inhibit proliferation, enhance apoptosis, induce a G0/G1 arrest, restrain T helper 1 type immune responses, and promote chemokine C-C motif ligand 20-mediated migration of human CD4^+^ T cells, which might be the mechanisms why anti-IL12p40 treatment presented efficacy in CD.

## Abstract 2

Supplemental Digital Content is available in the text

## INTRODUCTION

Inflammatory bowel diseases (IBDs), consisting of Crohn disease (CD) and ulcerative colitis (UC), are chronic debilitating disorders characterized by recurring episodes of relapsing and remitting inflammation of the gastrointestinal tract.^1^ Although the precise etiology of IBD has not yet been elucidated, a complex interaction between predisposing genes, environmental factors, and dysregulated mucosal immune responses to the commensal gut microbiome has been thought to make a great contribution to the disease pathogenesis.^2,3^

Among the numerous cytokines, interleukin (IL) 12 and IL23 were both considered as important inflammation mediators of innate and (or) adaptive immunity, and to have key role in driving intestinal inflammation.^4–7^ They had a common subunit named IL12p40, which was encoded by *IL12B* gene and was an important cytokine of IL12/23 pathway playing a crucial role in chronic intestinal inflammation.^8^ Genome-wide association studies (GWASs) and several case–control association studies have confirmed *IL12B* gene as susceptibility loci of both CD and UC across different racial and ethnic groups.^9–13^ In addition, some studies demonstrated that monoclonal antibodies (mAbs) against IL12p40 could abrogate established experimental colitis in mice.^14,15^ Furthermore, in a 36-week, double-blind, randomized, placebo-controlled phase 2b clinical trial, ustekinumab, which was a fully human IgG1κ IL12p40 mAb, had been proved to be effective in induction and maintenance therapy of refractory CD.^16^

Present studies have improved our understanding about the importance of IL12p40 in the pathogenesis of IBD; however, there were still 2 major issues to be clarified. The messenger RNA (mRNA) and protein expression of IL12B and its receptor IL12 receptor β 1 (IL12RB1) both locally (intestinal mucosal) and systemically (peripheral blood [PB]) had not been completely investigated, which was the basis of treatment using neutralization mAb. Although the proinflammatory effects of IL12p40 had been documented in the literature, the role of IL12p40 in the pathogenesis of IBD was still poorly understood; in other words, the mechanisms of efficacy of anti-IL12p40 treatment still remained unclear. In order to illustrate these questions, a series of experiments was designed.

## MATERIALS AND METHODS

### Study Subjects and Clinical Materials

All patients and healthy controls included in this study were recruited from Zhongnan Hospital of Wuhan University. The diagnosis of IBD was based on clinical, endoscopic, radiological, and histological criteria according to European Crohn's and Colitis Organisation guidelines.^17^ Patients complicated with other autoimmune diseases such as psoriasis, systemic lupus erythematosus, rheumatoid arthritis, and multiple sclerosis were excluded. Healthy controls routinely received an assessment for health status for those blood donors, and an additional evaluation by colonoscopy for those intestinal mucosa donors. All the biopsy specimens were taken from colonic mucosa. UC activity was evaluated using simple clinical colitis activity index (SCCAI), and active UC was defined as an SCCAI score >2.^18^ Activity of CD was assessed by best Crohn's Disease Activity Index (CDAI), and active CD was defined as a CDAI > 150.^19^ The detailed information of the included patients and healthy subjects was shown in Table 1 and supplemental methodology (see supplemental methodology, Supplemental Digital Content 1, http://links.lww.com/MD/A225, which illustrates the details of some experiments and statistical analysis).

**TABLE 1:**
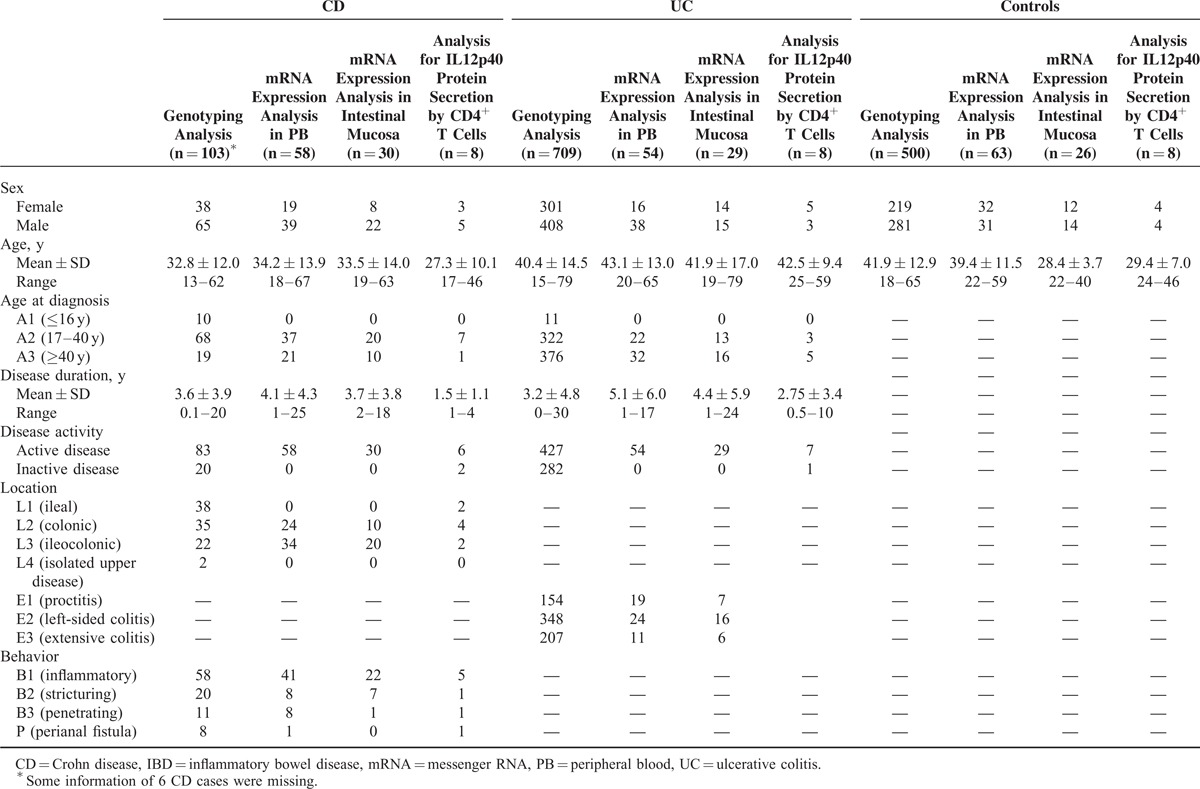
Demographics of IBD Patients and Controls Included in the Study

The study received approval from the ethics committee of the Zhongnan Hospital of Wuhan University. All participants signed an informed consent form.

### Real-Time PCR

The real-time polymerase chain reaction (PCR) reactions were performed as previously described.^20^ The detailed primers’ sequences were presented in supplemental methodology (ee supplemental methodology, Supplemental Digital Content 1, http://links.lww.com/MD/A225). A 2^−ΔΔCT^ method was used to identify the fold changes of patients group or experiments group relative to the control group.

### Western blotting

Western blot assays were also carried out as previously described.^20^ The detailed information of the primary antibodies was given as follows: antihuman IL12B (Epitomics, Burlingame, CA), antihuman IL12RB1 (Abcam, Cambridge, UK), antihuman IL12RB2 (Abcam), and antihuman Glyceraldehyde-3-Phosphate Dehydrogenase (Signalway Antibody, College Park, MD). A commercial horseradish peroxidase-conjugated secondary antibody was purchased from Abcam. Densitometrical analyses of the blots were performed using Quantity One software (Bio-Rad, Hercules, CA).

### Enzyme-Linked Immunosorbent Assay

For detection of soluble IL12p40 in PB, serum of IBD patients and healthy controls were separated. The serum IL12p40 levels were measured by a commercial enzyme-linked immunosorbent assay (ELISA) kit (Mabtech, Stockholm, Sweden) according to the manufacturer's instructions. The IL12p40 secreted by CD4^+^ T lymphocytes from IBD patients and healthy subjects was detected by the same way. In order to assess the effect of IL12p40 on cytokine secretion, supernatants from different groups were harvested after 48-hour culture of CD4^+^ T lymphocytes, and interferon-γ (IFN-γ), IL-4, IL-17, and IL10 were all determined by corresponding ELISA kit (Qiaoyi, GuangZhou, China).

### Isolation and Culture of CD4^+^ T Lymphocytes

Human PB mononuclear cells (PBMCs) were isolated using human lymphocyte separation medium (TBDscience, Tianjin, China) according to the manufacturer's instructions. CD4^+^ T cells were purified from PBMCs by a positive magnetic selection strategy using human CD4 MicroBeads (Miltenyi Biotec, Bergisch Gladbach, Germany). The purity of CD4^+^ T-cell population (CD3^+^ CD4^+^) was evaluated by flow cytometric analysis. The method of how to culture CD4^+^ T lymphocytes was described in supplemental methodology (see supplemental methodology, Supplemental Digital Content 1, http://links.lww.com/MD/A225).

### Flow Cytometric Analysis

Flow cytometry was used to perform the activation, apoptosis, and cell cycle distribution assays. The experiment details were presented in supplemental methodology (see supplemental methodology, Supplemental Digital Content 1, http://links.lww.com/MD/A225).

### Proliferation Assay

The impact of IL12p40 on the proliferation of CD4^+^ T cells was determined by 5-ethynyl-20-deoxyuridine (EdU) incorporation assay using the EdU assay kit (Ribobio, Guangzhou, China) according to the manufacturer's instructions. The experiment details were described in supplemental methodology (see supplemental methodology, Supplemental Digital Content 1, http://links.lww.com/MD/A225).

### Migration Assay

A 96-well Transwell system with a 5.0 μm polycarbonate membrane (Corning, NY) was used to perform the migration assay. A 150 μL culture medium with or without 500 ng/mL recombinant human chemokine C-C motif ligand 20 (CCL20), 200 ng/mL recombinant human chemokine C-C motif ligand 4 (CCL4), and 100 ng/mL recombinant human chemokine C-C motif ligand 5 (CCL5) was added into the lower chamber. After being cultured for 42 hours in the presence of IL12p40 neutralization antibody or mouse IgG2b isotype control antibody, 1 × 10^5^ cells were resuspended in 100 μL culture medium (accordingly with IL12p40 neutralization antibody or isotype control antibody) and added into the upper chamber. After 6 hours, cells in the lower chamber were counted using hemocytometers.

### Statistical Analysis

Numerical data were described as number of cases or percentages, and measurement data were presented as mean ± SD or median with interquartile range. The criteria of how to identify statistical methods is described in supplemental methodology (see supplemental methodology, Supplemental Digital Content 1, http://links.lww.com/MD/A225). Statistical analyses were carried out by using Statistical Product and Service Solutions (SPSS, Chicago, IL) Vision 17.0 for Windows. All calculated *P* values were 2-sided, and *P* < 0.05 was considered statistically significant.

## RESULTS

### Increased mRNA Expression of IL12p40 and its Receptor in PB and Mucosal Biopsy Specimens of IBD Patients

**FIGURE 1:**
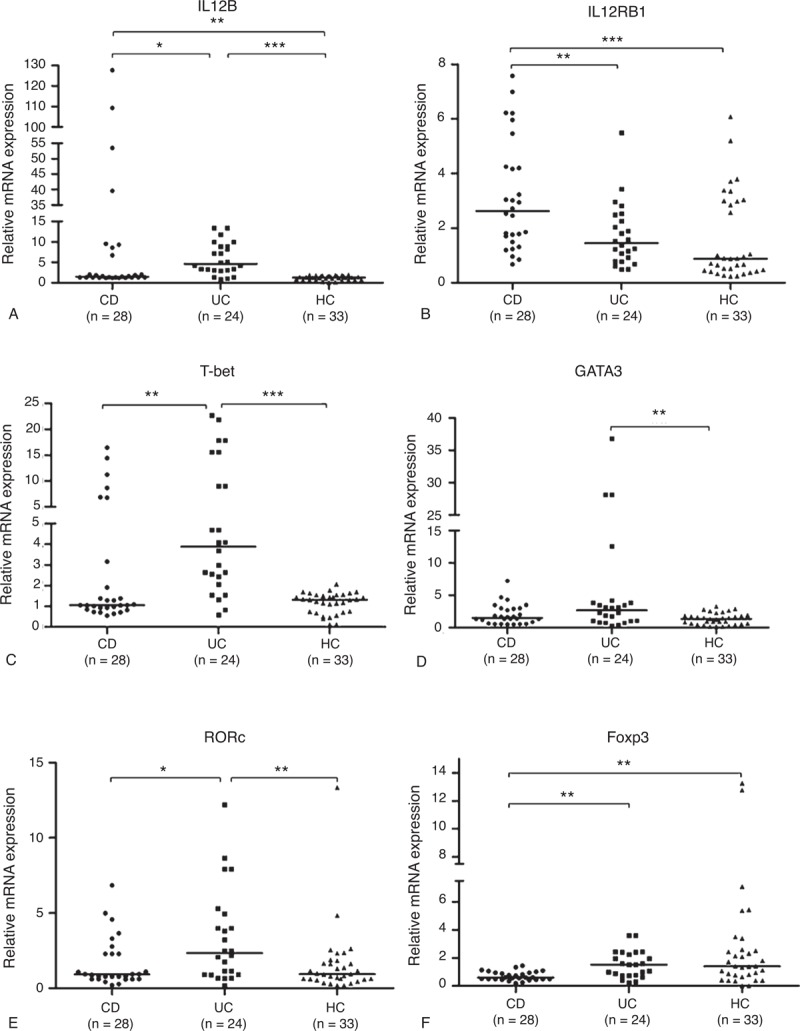
Analysis of mRNA expression of IL12B, IL12RB1, and transcript factors for Th cells in PB of IBD patients and HCs. (A) IL12B, (B) IL12RB1, (C) T-bet, (D) GATA3, (E) RORc, and (F) Foxp3 mRNA expressions were determined by real-time PCR from 28 CD patients, 24 UC patients, and 33 HCs (median values as horizontal bars in each group). All reactions were performed in 2 independent experiments for each gene with triplicate wells in each experiment. CD = Crohn disease, Foxp3 = forkhead box P3, GATA3 = GATA binding protein 3, HC = healthy control, IBD = inflammatory bowel disease, IL12RB1 = interleukin 12 receptor β 1, mRNA = messenger RNA, PB = peripheral blood, PCR = polymerase chain reaction, RORc = RAR-related orphan receptor C, Th = T helper, UC = ulcerative colitis. ^∗^*P < *0.05, ^∗∗^*P < *0.01, ^∗∗∗^*P < *0.001.

The expression of IL12B mRNA was elevated in PB of both active CD patients and active UC patients (*P* = 0.002 and 0.000, respectively), compared with the healthy subjects (Figure 1A). The UC patients had a significantly higher IL12B mRNA expression than CD patients (*P* = 0.033). The expression levels of IL12RB1, which could directly combine with IL12p40 subunit as a subunit of IL12R, were also increased in active IBD patients (Figure 1B), of which the CD cases expressed the greatest abundance, compared with UC cases and healthy controls (*P* = 0.006 and *P < *0.001, respectively). The difference of IL12RB1 mRNA expression between UC patients and healthy controls did not reach statistical significance (*P* = 0.12). T-bet, GATA binding protein 3 (GATA3), RAR-related orphan receptor C (RORc), and forkhead box P3 (Foxp3) were taken as the specific transcription factors of T helper 1 type (Th1) cell, T helper 2 type (Th2) cell, T helper 17 type (Th17) cell, and regulatory T (Treg) cell, respectively. The mRNA expressions of these transcription factors in PB were also detected (Figure 1C–F).

**FIGURE 2:**
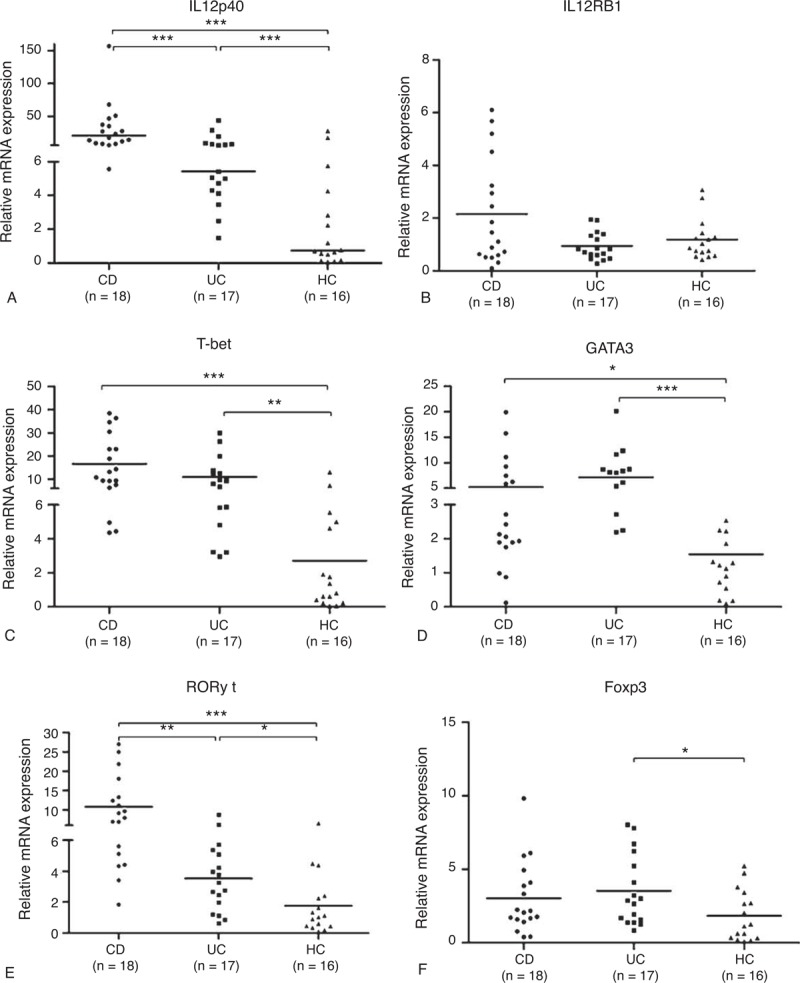
Analysis of mRNA expression of IL12B, IL12RB1, and transcript factors for Th cells in intestinal mucosa of IBD patients and HCs. (A) IL12B, (B) IL12RB1, (C) T-bet, (D) GATA3, (E) RORc, and (F) Foxp3 mRNA expressions were determined by real-time PCR from 18 CD patients, 17 UC patients, and 16 HCs (median values as horizontal bars in each group). All reactions were performed in 2 independent experiments for each gene with triplicate wells in each experiment. CD = Crohn disease, Foxp3 = forkhead box P3, GATA3 = GATA binding protein 3, HC = healthy control, IBD = inflammatory bowel disease, IL12RB1 = interleukin 12 receptor β 1, mRNA = messenger RNA, PCR = polymerase chain reaction, RORc = RAR-related orphan receptor C, Th = T helper, UC = ulcerative colitis. ^∗^*P < *0.05, ^∗∗^*P < *0.01, ^∗∗∗^*P < *0.001.

IL12B mRNA expression was also increased in the intestinal mucosa of IBD patients (Figure 2A). Unlike the expression pattern in PB, the elevation of IL12B mRNA expression was the most significant in CD patients, compared with the UC patients (*P < *0.001) and the healthy subjects (*P < *0.001). Meanwhile, the UC cases had an obvious increase of IL12B mRNA expression in contrast with the healthy controls (*P < *0.001). However, there was no statistical significance of IL12RB1 mRNA expression among the 3 groups (Figure 2B). In addition, there were also elevations of varying degrees for these transcription factors in mucosal specimens of IBD patients (Figure 2C–F).

To further identify whether *IL12B* genetic polymorphisms would affect the expression levels of IL12p40, we also investigated the polymorphisms of *IL12B* gene in 812 patients with IBD and 500 healthy controls in the Chinese population, and found that the polymorphisms of *IL12B* rs6887695 were associated with both CD and UC susceptibility (see supplemental data, Supplemental Digital Content 2, http://links.lww.com/MD/A225, which describes the demographics of IBD patients and controls included in the genotyping analysis and the associations between polymorphisms of *IL12B* and IBD susceptibility). Part of our published data revealed that the polymorphisms of *IL12B* rs6887695 were not associated with the serum IL12p40 protein level in UC patients,^17^ and such association was also determined in CD patients in this study (see Figure S1, Supplemental Digital Content 3, http://links.lww.com/MD/A225, which demonstrates the association between polymorphisms of IL12B rs6887695 and serum IL-12p40 levels in CD patients).

### A Significantly Positive Correlation Between IL12B mRNA Expression and T-Bet or RORγt mRNA Expression

Bivariate correlation analysis revealed a positive correlation between the expressions of IL12B mRNA and T-bet mRNA (*r* = 0.781, *P < *0.001) in PB of all CD patients, UC patients, and healthy controls (see table, Supplemental Digital Content 4, http://links.lww.com/MD/A225, which shows the results of bivariate correlation analysis of IL12B and IL12RB1 mRNA expressions with mRNA expression of specific transcription factors of CD4^+^ T-cell subtypes in PB), and also a strong positive correlation between the expressions of IL12B mRNA and T-bet mRNA (*r* = 0.807, *P < *0.001) or RORγt mRNA (*r* = 0.831, *P < *0.001) in the mucosal specimens of all the patients and healthy subjects (see table, Supplemental Digital Content 5, http://links.lww.com/MD/A225, which presents the results of bivariate correlation analysis of IL12B and IL12RB1 mRNA expressions with mRNA expression of specific transcription factors of CD4^+^ T-cell subtypes in intestinal mucosa), indicating that there might be a close connection between IL12B expression and the function of CD4^+^ T cell. However, it required more functional experiments to validate the speculation.

### Elevated IL12p40 Protein Expression in PB and Mucosal Biopsy Specimens of IBD Patients

**FIGURE 3:**
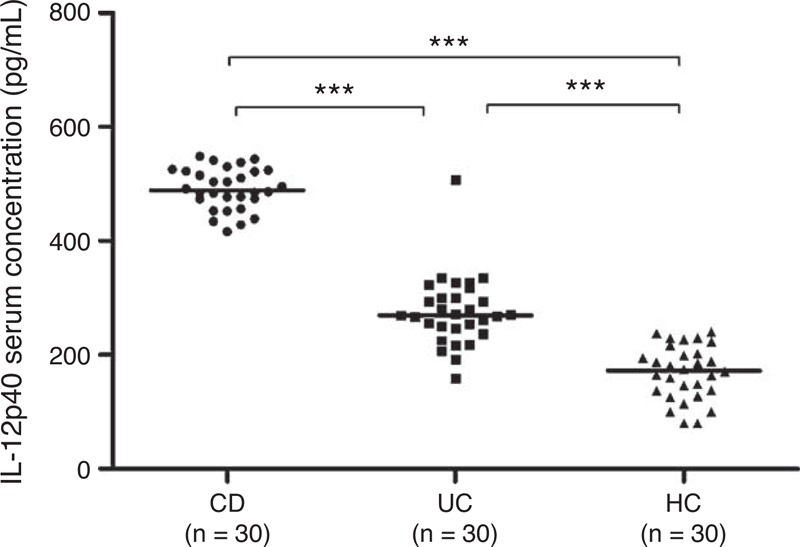
Serum IL12p40 levels were elevated in IBD patients and HCs. Serum IL12p40 levels were measured by ELISA from 30 CD patients, 30 UC patients, and 30 HCs. Tests were repeated for 2 times, with each independent assay in duplicate (mean as horizontal bars in each group). CD = Crohn disease, ELISA = enzyme-linked immunosorbent assay, HC = healthy control, IBD = inflammatory bowel disease, IL = interleukin, UC = ulcerative colitis. ^∗∗∗^*P < *0.001.

It was known from the peripheral serum ELISA that IL12p40 concentration was significantly higher in CD patients (491.3 ± 36.5 pg/mL) and UC patients (276.4 ± 61.8 pg/mL) than in healthy controls (168.9 ± 47.2 pg/mL), with both *P < *0.001 for CD and UC patients versus controls, respectively (Figure 3). Compared with UC patients, CD patients had a higher serum IL12p40 protein expression (*P < *0.001).

**FIGURE 4:**
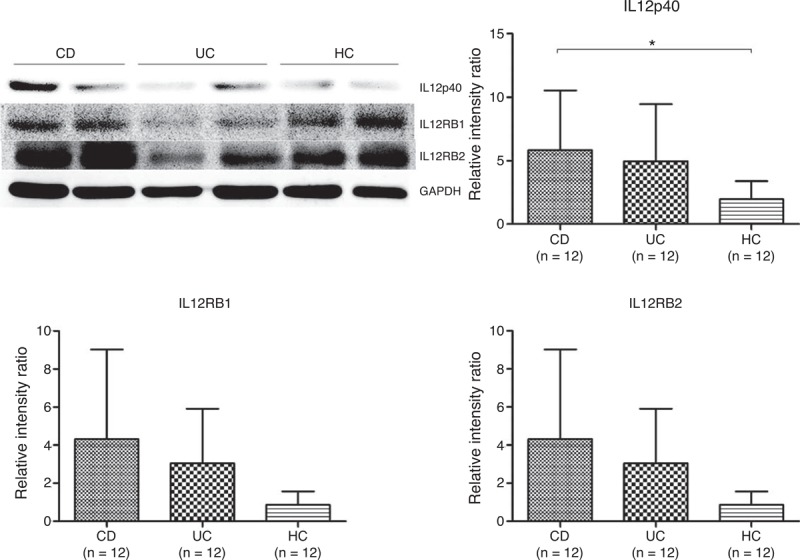
Analysis of protein expression levels of IL12p40, IL12RB1, and IL12RB2 in biopsy specimens from 12 CD patients, 12 UC patients, and 10 HCs. (A) Representative images of Western blotting; (B) IL12p40, (C) IL12RB1, and (D) IL12RB2 expression levels in each group. CD = Crohn disease, HC = healthy control, IL12RB1 = interleukin 12 receptor β 1, UC = ulcerative colitis. ^∗^*P < *0.05.

A Western blotting analysis was also performed to identify the expression levels of IL12p40 protein in the intestinal mucosa of IBD patients and healthy subjects (Figure 4A). Inflamed mucosal specimens of CD and UC patients both showed an elevated expression of IL12p40, IL12RB1, and IL12RB2 (another subunit of IL12R), but only statistical significance was reached by comparing the IL12p40 levels of intestinal mucosa between the CD patients and the healthy controls (*P* = 0.042, Figure 4B). All the protein levels of IL12p40, IL12RB1, and IL12RB2 in CD were higher than that in UC (Figure 4B–D).

### PB CD4^+^ T Cells From CD Patients Secreted High Levels of IL12p40 Protein

**FIGURE 5:**
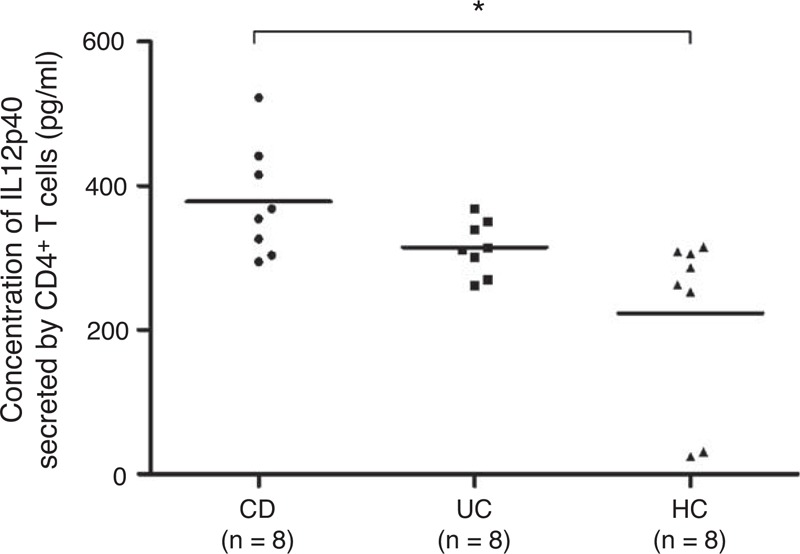
Analysis of IL12p40 secretion in the supernatants of human CD4^+^ T cells isolated from the PB of 8 CD patients, 8 UC patients, and 8 HCs after a 48-hour culture. Tests were repeated for 3 times, with each independent assay in triplicate (mean as horizontal bars in each group). CD = Crohn disease, HC = healthy control, IL = interleukin, PB = peripheral blood, UC = ulcerative colitis. ^∗^*P < *0.05.

To further investigate the direct association of IL12p40 expression with CD4^+^ T cells, PB-CD4^+^ T cells were isolated from IBD patients and healthy subjects, and were cultured as described above. The purity of isolated CD4^+^ T cells ranged from 99.0% to 99.3% by flow cytometry. After 48 hours of culture, the supernatants were harvested and assayed for IL12p40 secretion. Figure 5 demonstrated that CD4^+^ T cells from CD produced significantly higher levels of IL12p40 compared with controls (378.2 ± 77.5 pg/mL vs 223.2 ± 122.8 pg/mL, *P* = 0.027). Secretion levels of IL12p40 by UC CD4^+^ T cells (314.8 ± 37.4 pg/mL) were also higher than controls but lower than CD group. However, both the difference did not reach statistical significance.

### Neutralizing IL12p40 Secretion Did Not Affect the Activation of CD4^+^ T Cells

**FIGURE 6:**
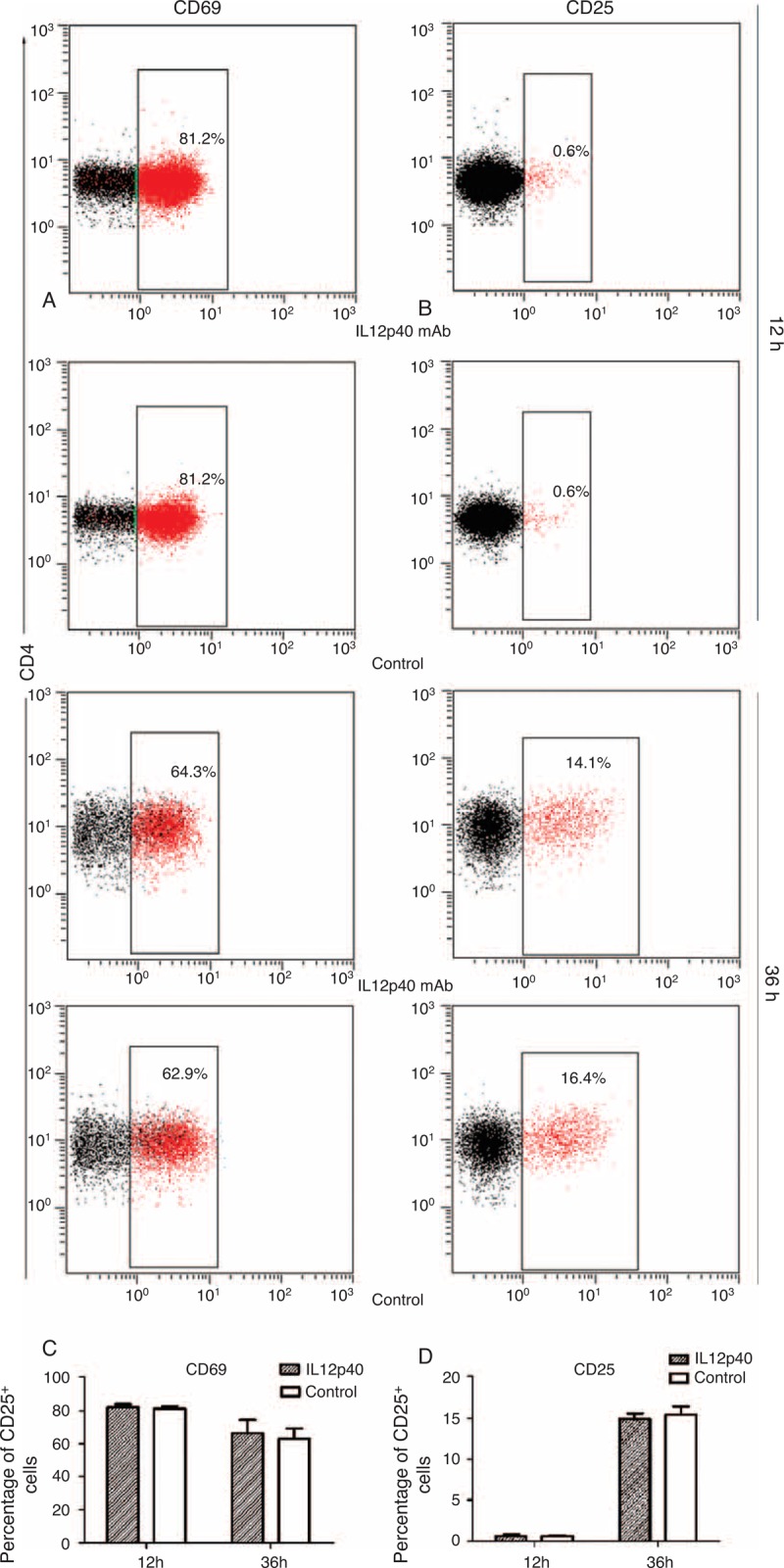
Impact of IL12p40 neutralization on activation of PB-CD4^+^ T cells after 12-hour and 36-hour cultures, respectively. Representative plots for each culture condition showing the expression of (A) CD69 and (B) CD25 within the CD4^+^ gate. The mean percentages of (C) CD69^+^ and (D) CD25^+^ cells were shown as histograms. Tests were repeated for 3 times, with each independent assay in duplicate. IL = interleukin, PB = peripheral blood.

After being incubated with IL12p40 neutralization antibody or mouse IgG2b isotype control antibody for 12 hours and 36 hours, respectively, CD4^+^ T cells were collected to detect the expression of CD69 and CD25 by flow cytometry, which were usually thought to be the activation markers of T cells.^21,22^ Our results revealed that IL12p40 neutralization had no influence on the CD4^+^ T-cell activation (Figure 6A–D).

### Neutralizing IL12p40 Secretion Inhibited the Proliferation of CD4^+^ T Cells

**FIGURE 7:**
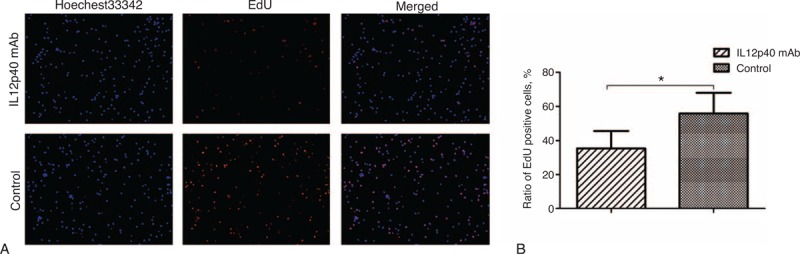
Impact of IL12p40 neutralization on proliferation of PB-CD4^+^ T cells after a 48-hour culture. (A) Representative images of immunofluorescence staining. EdU-labeled cells show red fluorescence and cell nuclei show blue fluorescence indicative of Hoechest33342 staining (magnification 100×). (B) The mean percentages of EdU-positive cells were shown as histograms. Tests were repeated for 3 times, with each independent assay in duplicate. EdU = 5-ethynyl-20-deoxyuridine, IL = interleukin, PB = peripheral blood. ^∗^*P < *0.05.

EdU incorporation assays revealed that the number of EdU positive cells in cells incubated with IL12p40 mAb was significantly reduced, as compared with cells treated with equivoluminal mouse IgG2b isotype control antibody (35.4% ± 10% vs 55.7% ± 12.2%, *P* = 0.021, Figure 7A and B), indicating that high concentration of IL12p40 promoted the proliferation of CD4^+^ T cells in vitro.

### Neutralizing IL12p40 Secretion Promoted the Apoptosis of CD4^+^ T Cells

**FIGURE 8:**
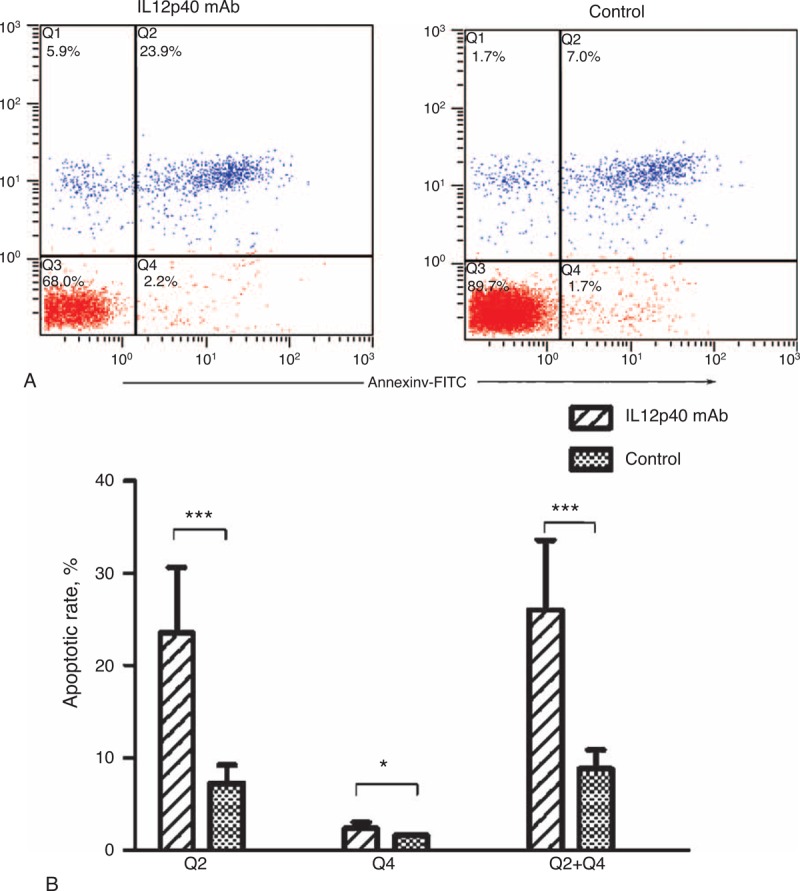
Impact of IL12p40 neutralization on apoptosis of PB-CD4^+^ T cells after a 48-hour culture. (A) Representative images of annexin V/PI dual staining detected by flow cytometry. (B) Apoptotic rates are expressed as mean ± SD in the corresponding bar graph. Tests were repeated for 3 times, with each independent assay in duplicate. IL = interleukin, PB = peripheral blood, PI = propidium iodide. ^∗^*P < *0.05, ^∗∗∗^*P < *0.001.

Different subpopulations were defined as follows: Q1, annexin V^−^/PI^+^, that is, necrotic cells; Q2, annexin V^+^/PI^+^, that is, late apoptotic cells; Q3, annexin V^−^/PI^−^, that is, live cells; Q4, annexin V^+^/PI^−^, that is, early apoptotic cells.^23,24^ In our study, an increased apoptotic rate was observed when cells were incubated with IL12p40 neutralization mAb for 48 hours. As shown in Figure 8, the ratios of early apoptotic cells (2.46% ± 0.56% vs 1.60% ± 0.11%, *P* = 0.025), late apoptotic cells (23.54% ± 7.08% vs 7.25% ± 1.98%, *P < *0.001), and total apoptotic cells (26.00% ± 7.59% vs 8.85% ± 2.00, *P < *0.001) were all significantly higher in the IL12p40 mAb group than that in the control group, indicating that IL12p40 played a key role in inhibiting the apoptosis of CD4^+^ T cells.

### Neutralizing IL12p40 Secretion Induced a G0/G1 Arrest of CD4^+^ T cells

**FIGURE 9:**
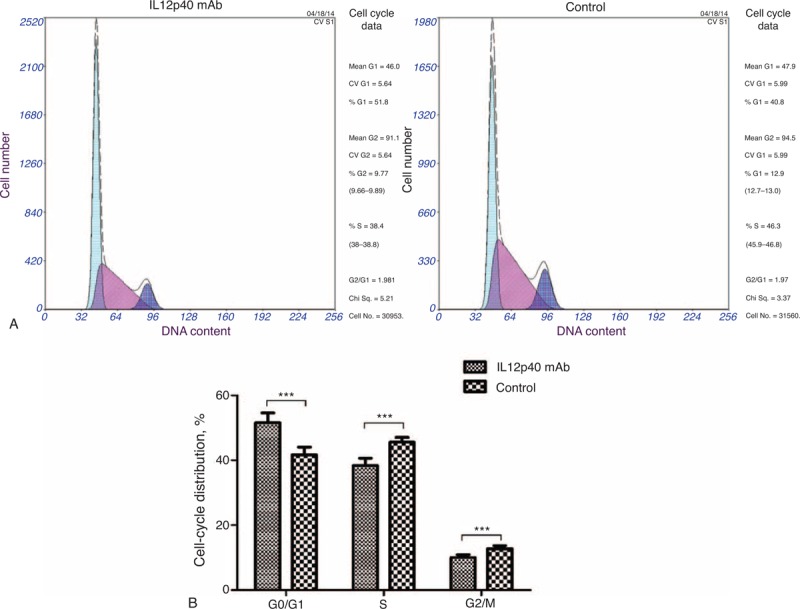
Impact of IL12p40 neutralization on the cell cycle distribution of PB-CD4^+^ T cells after a 48-hour culture. (A) Representative images of PI staining detected by flow cytometry and analyzed by Multicycle software (Fullerton, CA). (B) The percentages of the cell population in each phase (G1, S, and G2/M) of cell cycle were presented as mean ± SD in the corresponding bar graph. Tests were repeated for 3 times, with each independent assay in duplicate. IL = interleukin, PB = peripheral blood. ^∗∗∗^*P < *0.001.

Cell cycling was usually thought to be tightly associated with proliferation,^25,26^ so the impact of IL12p40 on cell cycling was also determined by flow cytometry. Although cells treated with mouse IgG2b isotype control antibody progressed through diverse cell cycle phases after activation by CD3 mAb and CD8 mAb, cells incubated with IL12p40 neutralization mAb displayed a totally different cell cycle pattern (Figure 9A and B). A higher frequency of cells at G0/G1 phases (51.6% ± 3.0% vs 41.7% ± 2.4%, *P < *0.001) and lower frequency of cells at S phase (38.4% ± 2.2% vs 45.6% ± 1.5%, *P < *0.001) and G2/M phases (10.0% ± 0.9% vs 12.8% ± 0.9%, *P < *0.001), respectively, were observed in the IL12p40 mAb group, indicating that IL12p40 was a crucial factor for proliferation of CD4^+^ T cells. This finding was consistent with the result of EdU incorporation assays.

### Neutralizing IL12p40 Secretion Downregulated Th1-Type Immune Response and Upregulated Th2-Type Immune Response

**FIGURE 10:**
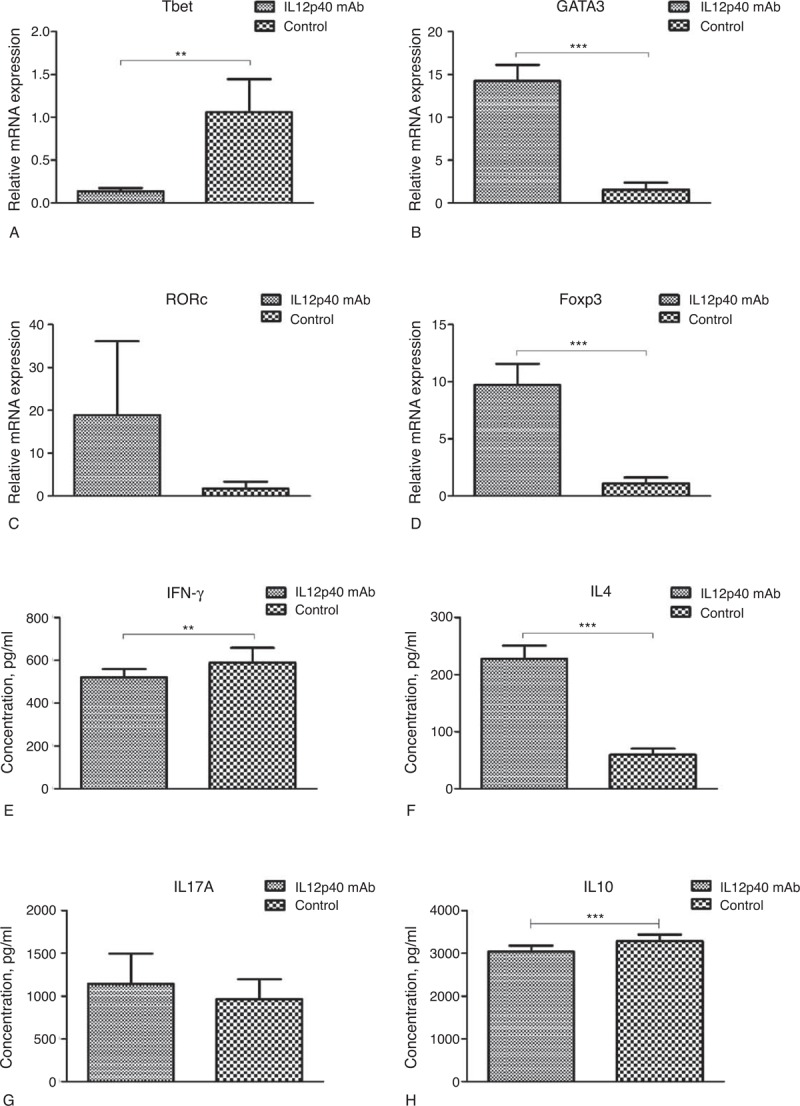
Impact of IL12p40 neutralization on differentiation of PB-CD4^+^ T cells after a 48-hour culture. The mRNA expression levels of (A) T-bet, (B) GATA3, (C) RORc, and (D) Foxp3 were detected by real-time PCR, and data were presented as mean ± SD in the corresponding bar graph. The cytokine secretion levels of (E) IFN-γ, (F) IL-4, (G) IL17A, and (H) IL10 were assayed by ELISA, and data were expressed as mean ± SD in the corresponding bar graph. Tests were repeated for 3 times, with each independent assay in triplicate. ELISA = enzyme-linked immunosorbent assay, Foxp3 = forkhead box P3, GATA3 = GATA binding protein 3, IFN = interferon, IL = interleukin, mRNA = messenger RNA, PB = peripheral blood, PCR = polymerase chain reaction, RORc = RAR-related orphan receptor C. ^∗∗^*P < *0.01, ^∗∗∗^*P < *0.001.

After a 48-hour culture, cells and supernatants were harvested. Cells were isolated to extract total RNA, thus used to perform real-time PCR to identify the mRNA expression of T-bet, GATA3, RORc, and Foxp3 (Figure 10A–D). Supernatants were assayed for IFN-γ, IL-4, IL17A, and IL10 (Figure 10E–H). Our results demonstrated that IL12p40 mAb incubation significantly reduced the T-bet mRNA expression (*P* = 0.002) and increased the GATA3 and Foxp3 mRNA expressions (both *P < *0.001), compared with the control group. The RORc mRNA expression was also elevated with IL12p40 mAb incubation, but the difference did not reach statistical significance (*P* = 0.059). IL12p40 mAb incubation also led to a different cytokine profile. IFN-γ secretion was significantly inhibited (519.8 ± 39.7 vs 589.2 ± 68.8 pg/mL, *P* = 0.009) and IL4 secretion was significantly enhanced (228.0 ± 22.5 vs 59.5 ± 10.9 pg/mL, *P < *0.001), which corresponded to the changes of T-bet and GATA3 mRNA expressions, respectively. Although a slight elevation of IL17A expression appeared in the IL12p40 mAb group, there was no statistical difference between both the groups (*P* = 0.17). In addition, an obvious decrease of IL10 secretion was observed when cells were incubated with IL12p40 mAb (3033.7 ± 143.7 vs 3291.6 ± 145.6 pg/mL, *P < *0.001).

### Neutralizing IL12p40 Secretion Enhanced Migration of CD4^+^ T Cells Toward CCL20

**FIGURE 11:**
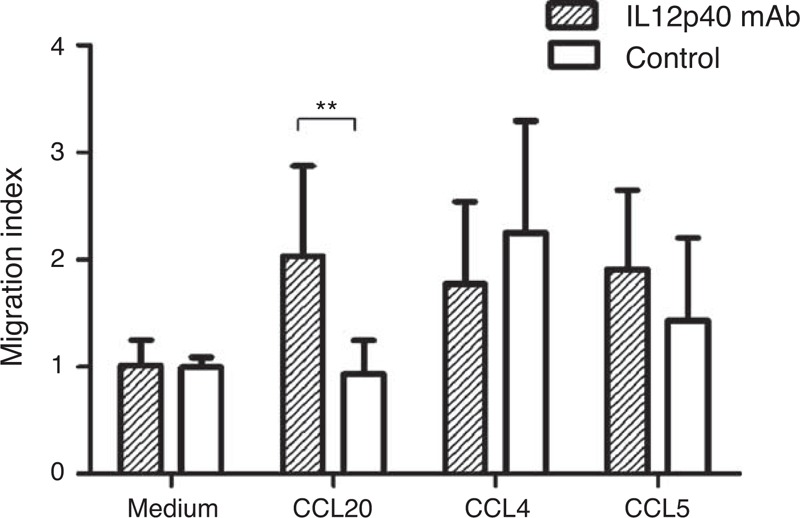
Impact of IL12p40 neutralization on migration of PB-CD4^+^ T cells toward different chemokines after a 48-hour culture. Migration index was calculated by dividing the numbers of migrated cells by the mean numbers of migrated cells seen in the medium-only wells of control group. Tests were repeated for 3 times, with each independent assay in triplicate. CCL = chemokine C-C motif ligand, IL = interleukin, PB = peripheral blood. ^∗∗^*P < *0.01.

Transwell assay revealed that cells incubated with 12p40 mAb showed significant migration to CCL20 (*P* = 0.004, Figure 11), compared with cells treated with mouse IgG2b isotype control antibody. A slight decrease of migration index for CCL4 and an increase for CCL5 were also observed in the experiment group, respectively, but both the differences did not reach statistical significance.

## DISCUSSION

Since IL12B had been identified as susceptibility loci for both CD and UC by GWASs, several validation studies were subsequently performed in different geographical and ethnic groups.^11–13,27–31^ Our results also found that single nucleotide polymorphism rs6887695 of IL12B was associated with both UC and CD pathogenesis in the Chinese Han population (Supplemental Digital Content 2, http://links.lww.com/MD/A225, supplemental data), but its association with CD was more pronounced. These genetic association studies revealed the possible contributions of *IL12B* gene in the pathogenesis of IBD, and gave key hints to the essential basis of subsequent functional studies.

Even before *IL12B* gene was identified as susceptibility loci of IBD, early studies had highlighted the role of both of IL12 and IL23 in the pathogenesis of IBD.^14,32–34^ IL12 was generally thought to play a vital role in the early immune modulation and Th1-mediated responses and was required for polarization of naïve T helper cells to the Th1 phenotype.^35–37^ IL23 was demonstrated to be a key inflammation mediator in Th17 responses.^38^ It could promote intestinal Th17 cell accumulation and drive intestinal T-cell proliferation.^7,15,39^ Confirmation of the expression levels of these cytokines was usually the first step of functional studies and the basis of targeted therapies. As important cytokines, their upregulation in IBD patients had been well reported.^40–43^ However, the expression levels of IL12p40 and its receptor had not been widely studied in IBD, especially in East-Asian population. Our previous study had even found elevated mRNA and protein expressions of IL12p40 both in PB and intestinal mucosa of UC patients.^20^ In this study, we comprehensively evaluated the expression of IL12p40 and its receptor IL12RB1 systematically and locally in both UC and CD patients and identified an upregulation of IL12p40 in IBD patients, indicating an important role of IL12p40 in IBD pathogenesis. Bivariate correlation analysis revealed that IL12B mRNA expression had a significantly positive correlation with T-bet or ROR-γt mRNA expression both at systematical and local levels, suggesting that IL12p40 might be tightly associated with Th1 or Th17 immune responses.

As an important part of the adaptive system, the role of CD4^+^ T cells in IBD onset and development had been concerned and affirmed for a long time.^44^ To validate the speculation from our correlation analysis, we isolated CD4^+^ T cells from CD patients, UC patients, and healthy controls respectively, and found that CD4^+^ T cells from CD patients secreted the most abundant IL12p40 protein. CD4^+^ T cells from UC patients also had a higher level of IL12p40 secretion than healthy subjects, but the difference did not reach statistical significance. Taken together, we hypothesized that IL12p40 might have an important impact on the functions of CD4^+^ T cells, which had rarely been studied.

Neutralization of IL12p40 had been proved to be effective in experimental colitis and CD patients.^14–16^ However, the mechanism of its efficacy remained unknown. In addition, to date, there were no randomized controlled trials to have evaluated whether IL12p40 neutralizing antibody was effective in UC patients. In our study, we first evaluated whether blocking IL12p40 protein would affect the activation of CD4^+^ T cells, but found no influence of IL12p40 signal on CD4^+^ T-cell activation. The number of T cells at the sites of inflammation was crucial for the maintenance and exacerbation of inflammation. Only a few studies had ever reported that anti-IL12 treatment could induce T-cell apoptosis in murine colitis model or human lamina propria mononuclear cells.^36,45–47^ However, no studies had ever evaluated whether extinction of IL12 signaling would promote the apoptosis of CD4^+^ T cells. In addition, the influence of IL12 signaling on CD4^+^ T-cell proliferation had also never been explored. Our apoptosis assay and proliferation assay revealed that anti-IL12p40 treatment promoted the apoptosis and inhibited the proliferation of CD4^+^ T cells in vitro, suggesting that a CD4^+^ T cell-mediated immune response would be attenuated. This might be the core of the mechanism that why anti-IL12p40 was effective in the therapies of autoimmune diseases such as psoriasis and CD. Furthermore, our cell cycle distribution analysis found that neutralizing IL12p40 led to a higher frequency of cells at G0/G1 phases and lower frequency of cells at S phase and G2/M phases, indicating a G0/G1 arrest of CD4^+^ T cells. This finding was consistent with the result of proliferation assay, and further confirmed the role of IL12p40 in promoting proliferation of CD4^+^ T cells. A Fas cell surface death receptor (FAS)-induced apoptosis was thought to take an important role in T-cell survival.^48^ By restraining the activity of caspase 3, IL12 could inhibit FAS-dependent apoptosis in murine experiments.^47^ In another murine colitis model, FAS-mediated T-cell apoptosis was also highlighted because that MRL/MpJ-lpr(fas) mice lacking Fas function developed colitis that responds poorly to anti-IL12 treatment.^45^ Whether CD4^+^ T-cell apoptosis induced by anti-IL12p40 treatment was also dependent on FAS-mediated signaling remained to be further verified.

CD was generally thought to be characterized as a Th1-mediated immune disorder, whereas UC was considered a Th2-mediated immune disease.^49^ In order to investigate the potential role of IL12p40 in the regulation of human CD4^+^ T-cell differentiation, the expression of 4 specific transcript factors mRNA and 4 selected cytokines were identified by real-time PCR and ELISA assay, respectively. Anti-IL12p40 treatment exhibited to markedly inhibit CD4^+^ T cell to express T-bet mRNA and IFN-γ production but promote them to express GATA3 and Foxp3 and IL4 production. The expression of ROR-γt and the secretion of IL17A were also enhanced by neutralizing IL12p40, however, the elevation was not obvious enough. Accordingly, we thought that enhanced IL12p40 expression upregulated the differentiation of human CD4^+^ T cells into Th1 cells characteristic of increased T-bet mRNA and IFN-γ production and inhibited them to differentiate into Th2 cells characteristic of decreased GATA3 mRNA and IL4 production, and Treg cells characteristic of decreased Foxp3 mRNA. Unexpectedly, IL10, an anti-inflammatory cytokine, was significantly reduced after blocking the IL12p40 signaling. Such decrease of IL10 secretion was also observed in patients with psoriasis receiving anti-IL12p40 therapies,^50^ but the exact mechanism was still unknown. Our study demonstrated that anti-IL12p40 could downregulate Th1 immune responses, which might be another important reason for remission of CD after anti-IL12p40 therapies. Our finding that elevated expression of IL12p40 of CD4^+^ T cells in vitro suppressed Th2 responses indicated IL12p40 might play different roles in the pathogenesis of UC and CD, respectively. However, it needed more studies in vivo to confirm the inference. Another important viewpoint that the early and late CDs presented distinct patterns of mucosal T-cell mediated immunoregulation had ever been proposed,^51,52^ the similar conclusion was also drawn from experimental colitis.^53^ Kugathasan et al^51^ found that early CD had a more strongly polarized Th1 type responses than late CD and pointed out that susceptibility to IL12-mediated modulation was strongly dependent on the stage of CD. In a mice model, the researchers also found that IL12 played a pivotal role only in early colitis but not in late colitis.^53^

Finally, a Transwell system was used to determine the migration of human CD4^+^ T cells toward different chemokines including CCL4, CCL5, and CCL20. An obvious migration mediated by CCL20 was observed in CD4^+^ T cells receiving anti-IL12p40 treatment. Cook et al^54^ had even found that Foxp3^+^, but not Foxp3^−^, CD4^+^ cells from *Helicobacter pylori*-infected mice migrated to recombinant CCL20 in vitro. Combined with our finding that Foxp3 mRNA expression of CD4^+^ T cells was elevated after anti-IL12p40 treatment, we speculated that CCL20-mediated migration of Foxp3^+^ Treg cells toward inflammation sites might enhance the anti-inflammatory effect. However, this speculation remained to be confirmed by further vivo experiments.

In summary, the expression of IL12p40 was elevated both at mRNA and protein levels systematically and locally in IBD patients but more significantly in CD patients. Furthermore, our findings revealed that IL12p40 signaling had a key impact on the functions of human CD4^+^ T cells, which might be an important part of IBD pathogenesis and the mechanisms why anti-IL12p40 treatment presented efficacy in CD.

## ACKNOWLEDGMENTS

We are grateful to the patients and healthy volunteers for their kind assistance with donating blood and biopsy samples. We also thank Ping Zhang for her statistical expertise.
